# Emerging Trends in Machine Learning for Signal Processing

**DOI:** 10.1155/2017/6521367

**Published:** 2017-11-19

**Authors:** George A. Papakostas, Konstantinos I. Diamantaras, Francesco A. N. Palmieri

**Affiliations:** ^1^Humain-Lab, Eastern Macedonia and Thrace Institute of Technology, Kavala, Greece; ^2^TEI of Thessaloniki, Sindos, Greece; ^3^Seconda Università di Napoli (SUN), Aversa, Italy

Recently, there is an increasing interest in developing “smart” devices and systems able to interact with their environment, for example, Internet of Things and Human-Machine Interfaces. The term “smart” is used to describe a set of advanced functionalities implemented utilizing sophisticated computational intelligence (CI) algorithms. Machine learning (ML) constitutes an important area of CI dealing with the ability of computers/machines to learn through knowledge representation, processing, and storing. ML offers solutions to difficult engineering problems, in a similar way to the humans' brain processing. Moreover, considering the large amount and diversity of data (image, video, time series, 1D signals, text, etc.) massively generated and stored by modern “smart” systems, the need for efficient ML algorithms in terms of accuracy and speed become increasingly important. In the light of this rapid development of machine learning tools, this special issue focuses on recent trends in applying ML methodologies for processing signals coming from any source.

One paper of this issue addresses the diagnosis of pathological brain images using well known image processing tools (Wiener filtering, 2D-DWT, and Probabilistic PCA) and machine learning models (Random Subspace Ensemble (RSE), *K*-nearest neighbors). The proposed methodology was compared with 21 state-of-the-art algorithms, in terms of accuracy, sensitivity, and specificity for four datasets.

Another paper presents a novel active semisupervised Convolutional Neural Network (CNN) algorithm able to recognize SAR images without the need for a large number of labeled samples in the training phase. Initially, the method applies active learning for the labeled data and then a semisupervised regularization process is designed for the remaining unlabeled data.

One paper is on the application of intuitionistic fuzzy Intercriteria Analysis on reducing the number of input parameters of a Multilayer Perceptron neural network. This will allow the reduction of the weight matrices, as well as the implementation of the neural network in limited hardware, thus this will save time and resources in training.

Another paper deals with the face recognition problem. For this purpose a patch-based Principal Component Analysis (PCA) method is proposed that utilizes the local spatial information enclosed in face image patches. Extensive experiments on two benchmark face datasets showed outperformance of the proposed method against several PCA-based similar algorithms.

One of the papers explores the use of recorded deep brain local field potentials (LFPs) for robust movement decoding of Parkinson's Disease (PD) and Dystonia patients. A novel ensemble classifier was proposed for accurate prediction of finger movement and its forthcoming laterality. The ensemble classifier consists of three base neural network classifiers, namely, feed-forward, radial-basis, and probabilistic neural networks, while the majority-voting rule was used to fuse the decisions of three base classifiers to generate the final decision.

Another paper proposes a novel personal verification system based on the likelihood ratio test for fusion of match scores from multiple biometric matchers (face, fingerprint, hand shape, and palm print). Zernike moments are used as multimodal features during the matching phase and after the fusion of the match scores a Gaussian Mixture Model estimates the genuine and impostor densities of match scores for personal verification.

One paper explores the applicability of deep learning techniques for identifying unexpected behavior in ship plot and track patterns, as captured by an Over-The-Horizon (OTH) radar. The proposed methodology exploits the nonlinear mapping capabilities of deep stacked autoencoders in combination with density based clustering. A detailed comparative experimental evaluation of the approach shows promising results.

*George A. Papakostas* *Konstantinos I. Diamantaras* *Francesco A. N. Palmieri*

